# Exploring the Impact of Digital Health Tools in Enhancing Quality of Life and Psychological Adjustment in Long-term Blood Donors: A Cross-Sectional Study

**DOI:** 10.7759/cureus.75123

**Published:** 2024-12-04

**Authors:** Turki M Alanzi, Wejdan Arif, Nader Aljohani, Abdulaziz Jabali, Mohammed Junainah, Emad Aldeen Mohamed, Turki Hamdi, Nawaf Mansour, Nawaf Badawood, Saeed Alghamdi, Dalia Alanazi, Nouf Alanzi, Nehal Alqurashi

**Affiliations:** 1 Health Information Management Technology, Imam Abdulrahman Bin Faisal University, Dammam, SAU; 2 Radiological Sciences, College of Applied Medical Sciences, King Saud University, Riyadh, SAU; 3 College of Medicine, King Abdulaziz University, Jeddah, SAU; 4 Pharmaceutical Care, King Abdulaziz Medical City, Jeddah, SAU; 5 Spine Surgery, Prince Sultan Military Medical City, Riyadh, SAU; 6 Medical Laboratory Technology, College of Applied Medical Sciences, University of Tabuk, Tabuk, SAU; 7 Clinical Laboratory Sciences, Jouf University, Sakakah, SAU; 8 General Practice, Eradah And Mental Health Complex, Taif, SAU

**Keywords:** anxiety, blood donation, digital health tools, donors, psychological adjustment, quality of life

## Abstract

Background: Blood donation plays a critical role in public health, yet long-term donors (donating at least twice per year, for at least three years) often face challenges related to psychological adjustment and quality of life. Digital health tools could offer innovative solutions to address these issues by providing personalized support, tracking, and mental health interventions.

Aim: This study investigates the impact of digital health tools on the quality of life (QoL) and psychological adjustment of long-term blood donors in Saudi Arabia, with attention to demographic factors influencing engagement and perceptions.

Methods: A cross-sectional survey was conducted among 498 long-term blood donors aged 18-60, recruited from blood donation centers across Saudi Arabia. Participants were recruited through social media platforms, university networks, and professional organizations to ensure a diverse sample in terms of background, long-term donation history (more than 10 times), and daily digital usage habits. Participants completed an online questionnaire incorporating the WHO Quality of Life-Brief Version (WHOQOL-BREF) and Coping Orientation to Problems Experienced Inventory (Brief-COPE) inventory to assess QoL and coping mechanisms. Descriptive statistics, two-sample t-tests, and ANOVA analyses were performed to examine differences in perceptions based on age, gender, and educational level.

Results: The findings indicate a generally positive perception of digital health tools, with mean QoL scores for self-distraction (Mean ± SD: 3.33 ± 0.97), active coping (3.29 ± 0.97), and psychological well-being (3.31 ± 0.82). Significant differences were observed across age groups, with older participants reporting higher perceived benefits ( p < .0001). Gender differences were also significant, with females showing higher mean scores in psychological adjustment factors such as active coping (3.75 ± 0.62) compared to males (2.8 ± 0.81), p < .0001.

Conclusion: Digital health tools are perceived as beneficial for supporting QoL and psychological adjustment among blood donors, particularly for emotional and health management. Demographic factors such as age, gender, and education play a significant role in shaping these perceptions, highlighting the need for tailored interventions. Further research should explore longitudinal impacts to inform the design of more effective, culturally aligned digital health solutions for donor support.

## Introduction

The integration of digital health tools into healthcare has transformed how individuals manage their health, providing opportunities for improved quality of life (QoL) and psychological adjustment. These tools encompass mobile health applications, wearable devices, and telemedicine platforms that enable users to monitor health metrics, access personalized feedback, and engage in health-promoting behaviors [1-4]. While much of the focus has been on managing chronic conditions such as diabetes and hypertension [5-9], there is increasing recognition of their potential to address the unique challenges faced by specific populations, including long-term blood donors.

Blood donation, a critical public health activity, relies heavily on the sustained contributions of long-term donors. Long-term blood donors, defined in this study as individuals who donate blood at least twice per year for a minimum of three years, play a crucial role in maintaining a stable and reliable blood supply. These donors are often highly committed to the practice, driven by altruism and a sense of social responsibility. However, these individuals often face both physical challenges, such as fatigue, iron depletion, and dizziness [10-12], and psychological concerns, including stress, anxiety, and societal perceptions about repeated donations [13-15]. These challenges can disrupt social relationships, impact emotional well-being, and ultimately affect donors' long-term commitment [16,17].

Within Saudi Arabia, maintaining a stable blood supply is essential, yet achieving this goal is complicated by a geographically dispersed population, varying health literacy levels, and cultural perceptions around blood donation [18-22]. Digital health tools offer a promising avenue for addressing these issues by improving donor engagement, providing health monitoring, and supporting psychological well-being. While studies have highlighted the benefits of digital platforms in healthcare, there is a lack of localized research exploring their application among Saudi blood donors. This study aims to fill this gap by examining how digital health tools can improve both the QoL and psychological adjustment of long-term donors in Saudi Arabia, emphasizing the cultural and healthcare system context.

Background

Research demonstrates the effectiveness of digital health tools in managing chronic health conditions. For example, Debon et al. showed that mobile applications for individuals with hypertension enhanced their ability to monitor blood pressure and make informed decisions, contributing to improved QoL [23]. Similarly, wearable devices tracking physical activity, heart rate, and sleep patterns have been linked to better disease management and psychological outcomes in individuals with chronic conditions [24-27]. These tools enable real-time monitoring, personalized feedback, and tailored interventions, making healthcare more accessible and proactive.

In contrast, research on the use of digital health tools specifically for blood donors remains limited. Although some parallels exist between the needs of long-term donors and individuals with chronic conditions such as the necessity for ongoing health monitoring and strategies for psychological support, the challenges faced by donors are unique [28-29]. Psychological stressors, including anxiety about health risks and societal stigmas, can discourage continued participation in blood donation programs [30-32]. Long-term donors must navigate these barriers to sustain their commitment, which makes targeted interventions essential [33-38]. Studies have acknowledged the potential role of digital tools in supporting blood donors. For instance, gamified mobile applications have been used to educate donors, improve physical health outcomes, and foster engagement [39]. Telemedicine platforms offering remote consultations have provided continuous support, enhancing adherence to donation schedules and overall well-being [40]. However, these efforts predominantly focus on physical health and do not adequately address psychological adjustment or QoL, particularly within specific cultural contexts.

In Saudi Arabia, where cultural and systemic factors significantly influence blood donation practices, localized research is crucial. The unique societal perceptions, coupled with the rapid modernization of the healthcare system, present an opportunity to explore tailored solutions. By leveraging digital health tools, there is potential to address the psychological and physical challenges faced by long-term blood donors while simultaneously promoting health literacy and engagement.

Objectives

This study aims to investigate the impact of digital health tools in enhancing the QoL and psychological adjustment of long-term blood donors in Saudi Arabia. The research will focus on the development and application of culturally relevant digital interventions, assessing their effectiveness in mitigating physical and emotional challenges and supporting sustained donor participation.

## Materials and methods

Study settings and participants

An online cross-sectional survey design was adopted in this study conducted in Saudi Arabia, targeting long-term blood donors who have participated in at least 10 donation events over the past five years at blood banks and healthcare facilities. The study encompassed both urban and rural settings to capture the variability in access to healthcare and digital tools, as well as differing cultural attitudes toward blood donation. Participants were selected from key regions, including Riyadh, Jeddah, and the Eastern Province, representing diverse socioeconomic backgrounds, levels of health literacy, and technological adoption. This approach ensured the representation of donors with varying experiences in terms of healthcare access, physical and psychological challenges, and their use of digital health tools, thereby providing a comprehensive understanding of the long-term blood donor population in Saudi Arabia. 

The inclusion criteria for participants were: individuals aged 18-60 years, who have been regular blood donors (donating at least twice per year) for at least 3 years, and who voluntarily agreed to participate in the study. The age range of 18-60 years was selected to align with international blood donation guidelines, which typically recommend eligibility between these ages for ensuring donor safety and adequate physical capacity for donation [41,42]. Additionally, this range encompasses the majority of active donors, as older populations often face age-related health issues that may limit donation frequency [43].

The requirement of at least three years of regular blood donation was set to ensure that participants had sufficient experience as long-term donors, allowing the study to focus on those who are familiar with the physical and psychological impacts of repeated donations. Prior research highlights that donors with over three years of consistent participation are more likely to experience long-term effects and have established donation habits [44,45].

Exclusion criteria included individuals with physical or mental health conditions that could interfere with their ability to complete the survey or participate in the digital health tools usage assessment. This exclusion was based on ethical considerations to avoid placing additional burdens on individuals whose health conditions might limit their ability to engage with the study interventions or skew results due to unrelated health factors. Similar studies on digital health tools emphasize the importance of excluding individuals with confounding health conditions to maintain study validity and participant safety [46,47].

Sampling and recruitment

The sampling strategy employed in this study was purposive [48], aiming to recruit long-term blood donors with extensive blood donation experience, defined as individuals who have donated regularly for at least three years. Purposive sampling was chosen due to the need to focus on a specific subgroup of blood donors who could provide in-depth insights into the physical, psychological, and behavioral aspects of long-term blood donation. This approach allows researchers to ensure that the participants align closely with the study’s objectives, which require experience with repeated donation and potential interactions with digital health tools. While purposive sampling has the potential to introduce selection bias, it is particularly suitable for exploratory research where the goal is to generate insights from a targeted population rather than to achieve statistical generalizability [49]. By selecting participants across diverse demographics, including age, gender, and geographic location (urban and rural regions within Saudi Arabia), the study aimed to mitigate this limitation and ensure the representation of a wide range of donor experiences. This approach is supported by previous studies in healthcare research, where purposive sampling has been successfully employed to explore the nuanced experiences of specific populations [50].

Participants were selected from blood donation centers in key urban and rural areas, ensuring the sample's diversity and representativeness. Recruitment took place in collaboration with local hospitals, blood banks, and healthcare providers, who assisted in reaching potential participants. Additionally, healthcare professionals at the donation centers provided verbal invitations to eligible donors during their routine visits. Interested individuals were then directed to the study's online platform or to on-site recruitment staff for further details about participation. The target sample size is approximately 500 participants (based on an estimated sample of 383 calculated using Cochran’s formula [51]), distributed proportionally across the selected regions. This sample size is designed to provide sufficient statistical power for analyzing variations in public perceptions, behaviors, and attitudes toward air quality and respiratory health across different demographic and environmental contexts in Saudi Arabia.

A total of 523 participants were included in the study; however, 25 participants did not complete the full survey, leading to a final sample of 498 participants.

Instruments

To measure the quality of life and psychological adjustment of the participants, two validated instruments were employed: the WHO Quality of Life-Brief Version (WHOQOL-BREF) [52] and the Coping Orientation to Problems Experienced Inventory (Brief-COPE) inventory [53]. WHOQOL-BREF is a widely recognized instrument developed by the WHO to assess the QoL across multiple dimensions. It consists of 26 items that measure four domains: physical health, psychological well-being, social relationships, and environmental factors. The WHOQOL-BREF is particularly suitable for this study as it provides a comprehensive evaluation of how digital health tools may impact various aspects of the participants’ lives, from their physical health and psychological state to their social interactions and environmental satisfaction. Questions are rated on a five-point Likert scale, with higher scores indicating better quality of life. This instrument's broad scope makes it ideal for capturing the multifaceted effects of digital health tools on long-term blood donors. The Brief COPE scale is a validated tool used to assess how individuals cope with stress and challenges in their lives. It is a shortened version of the COPE inventory and includes 28 items that measure 14 different coping strategies, such as active coping, planning, positive reframing, and seeking social support. The Brief COPE is particularly relevant for this study as it allows researchers to evaluate the coping mechanisms employed by long-term blood donors when managing the psychological demands associated with regular blood donation. By analyzing the use of digital health tools in relation to these coping strategies, the study aims to determine whether these tools facilitate more effective coping and contribute to better psychological adjustment.

These tools were adapted to suit the cultural and linguistic context of Saudi Arabia. The adaptation process included translation into Arabic using a forward-translation and back-translation method to ensure semantic equivalence, as recommended for cross-cultural research [54]. Additionally, a panel of bilingual experts in psychology and public health reviewed the scale for cultural relevance and clarity, making adjustments to items that may not align with local norms or experiences. For example, coping strategies involving social support were contextualized to reflect the importance of family and community in Saudi culture. A pilot test was conducted with a small sample of Saudi blood donors (n = 30) to assess the tool's reliability and validity in the target population. The data collected from this pilot study were analyzed, and Cronbach's alpha coefficient for all items was calculated. The coefficient exceeded 0.7, indicating robust internal consistency and reliability of the questionnaire [55]. Feedback from the pilot study was used to refine the language and ensure the scale accurately captured the coping mechanisms relevant to this group.

Data collection

Data for this study were collected over four weeks using a structured survey questionnaire, available in both English and Arabic. The questionnaire was distributed exclusively through online platforms to ensure broad accessibility and convenience for participants across various regions in Saudi Arabia, including urban, suburban, and rural areas. The online distribution leveraged social media, email networks, and community forums to reach a diverse population. This digital approach facilitated the collection of comprehensive data on public perceptions and practices related to air quality and respiratory health while ensuring participants could complete the survey at their convenience.

Data analysis

To achieve the study's objectives, the data were analyzed using the IBM SPSS Statistics for Windows, Version 24.0 (Released 2016; IBM Corp; Armonk, New York, United States). Descriptive statistics, including means and standard deviations, were employed to present the demographic characteristics of the participants. Additionally, a two-sample t-test with unequal variances and a one-way ANOVA were conducted to analyze the data further.

Ethical considerations

The study received approval from the Research Ethics Committee at Imam Abdulrahman Bin Faisal University (approval number: IRB-2024-03-606). Informed consent was obtained from all participants prior to their involvement, with clear explanations provided regarding the study's purpose, procedures, and potential risks. To address concerns about discomfort with sensitive survey questions, participants were informed of their right to skip any question or withdraw from the study at any time without any repercussions. Additionally, the survey design avoided intrusive or overly personal questions, focusing instead on general coping mechanisms, digital health tool usage, and overall well-being.

To ensure robust confidentiality and data security, anonymization was carried out through the removal of all directly identifiable data such as names, contact information, and IP addresses from the dataset prior to analysis. A unique alphanumeric code was assigned to each participant to enable data tracking while preserving anonymity. Data were securely stored on encrypted servers accessible only to authorized research personnel. Furthermore, the study adhered to ethical guidelines for research involving digital health tools, ensuring that privacy concerns related to these tools were clearly addressed in the consent process.

By implementing these measures, the study prioritized participant safety, confidentiality, and trust, aligning with ethical research standards while minimizing potential risks associated with privacy or sensitive questions.

## Results

A total of 523 participants were included in the study; however, 25 participants did not complete the full survey, leading to a final sample of 498 participants. As shown in Table 1, the demographic data revealed a balanced gender distribution among participants, with a slight predominance of females (50.8%) over males (49.2%). The age distribution highlighted a younger sample, with 34.7% of participants aged 18-30, followed by 22.5% in the 31-40 age group. Middle-aged groups (41-50 years and 51-60 years) represented a combined 34.2% of the sample, while only 8.6% of participants were over 60, suggesting that the findings might be more relevant for younger and middle-aged demographics. Education levels showed diverse representation: participants with a diploma were the largest group (27.3%), followed by those with primary or secondary education (20.7%). Bachelor's degree holders constituted 20.3%, while those with a Master’s degree or higher represented 17.1%, and uneducated participants made up 14.7%. This range across educational levels may have contributed to varied perspectives within the sample, supporting generalizability across different educational backgrounds.

**Table 1:** Participants demographics (N=498)

Factors	Variables	Frequency (Percentage)
Gender	Male	245 (49.2%)
	Female	253 (50.8%)
Age (in years)	18-30	173 (34.7%)
	31-40	112 (22.5%)
	41-50	86 (17.3%)
	51-60	84 (16.9%)
	>60	43 (8.6%)
Occupation	Uneducated	73 (14.7%)
	Primary/Secondary education	103 (20.7%)
	Diploma	136 (27.3%)
	Bachelor’s degree	101 (20.3%)
	Master’s degree or higher	85 (17.1%)

Table 2 provides an overview of participants' perceptions regarding the impact of digital health tools on their QoL and psychological adjustment. Across all factors, the mean scores generally hovered around 3.25-3.36, suggesting a moderate to positive perception of these tools. Self-distraction and denial received the highest mean scores ± SD (3.36 ± 0.97 and 3.33 ± 0.98, respectively), indicating that participants perceive these tools as particularly helpful in managing stress or emotional challenges. On the other hand, substance use and venting had the lowest mean scores (3.21 and 3.24, respectively), suggesting that while digital health tools were still perceived positively, they may have less of an impact on managing these specific behaviors. Active coping and positive reframing scored relatively high as well, suggesting that participants viewed digital tools as useful in fostering adaptive coping strategies.

**Table 2:** Participants perceptions on the impact of digital health tools on quality of life and psychological adjustments SD: Standard deviation

Factors		Mean ± SD
Quality of Life	Self-distraction	3.33 ± 0.97
	Active coping	3.29 ± 0.97
	Denial	3.36 ± 0.98
	Substance use	3.26 ± 1.04
	Use of emotional support	3.27 ± 1
	Use of instrumental support	3.29 ± 1.02
	Behavioral disengagement	3.24 ± 1
	Venting	3.21 ± 0.95
	Positive reframing	3.32 ± 0.99
	Planning	3.27 ± 0.98
	Humor	3.26 ± 1.04
	Acceptance	3.23 ± 0.99
	Religion	3.27 ± 1.02
	Self-blame	3.32 ± 0.99
Psychological Adjustment	Physical health	3.3 ± 0.8
	Psychological well-being	3.31 ± 0.82
	Social relationships	3.27 ± 0.88
	Environment	3.29 ± 0.8

Regarding psychological adjustment, participants rated physical health and psychological well-being highly (3.30 ± 1.04 and 3.31 ± 0.95, respectively), signifying the positive role of digital health tools in improving overall health and mental well-being. Social relationships and environmental factors were also perceived positively, with mean scores of 3.27 ± 0.88 and 3.29 ± 0.80, respectively, indicating that participants felt these tools support not only individual well-being but also social and environmental contexts. Overall, the data suggests a broadly favorable perception of digital health tools in enhancing both quality of life and psychological adjustment, with some factors, such as denial and self-distraction, standing out as particularly beneficial.

The one-way ANOVA of the data (Table 3) revealed meaningful insights into how long-term blood donors' perceptions of digital health tools vary across different age groups. The significant differences observed suggest that older participants tend to perceive digital health tools as more beneficial for their psychological adjustment and QoL compared to younger donors. This may be attributed to a variety of factors, such as the greater experience and possibly more pronounced health concerns among older individuals, which could make them more receptive to health interventions. Conversely, younger blood donors may have different health-related priorities or expectations, potentially explaining their lower ratings across most variables.

**Table 3:** One-way ANOVA results assessing difference between participants' perceptions on the impact of digital health tools on quality of life and psychological adjustments of long-term blood donors - based on age * Statistically significant difference at .05 confidence interval

Factors	Age (in years)	N	Mean ± SD	p-value
Self-distraction	18-30	173	2.86 ± 0.95	< .0001*
	31-40	112	3.61 ± 0.88	
	41-50	86	3.83 ± 0.92	
	51-60	84	3.35 ± 0.85	
	>60	43	3.42 ± 0.82	
Active coping	18-30	173	2.82 ± 0.92	< .0001*
	31-40	112	3.62 ± 0.92	
	41-50	86	3.78 ± 0.83	
	51-60	84	3.33 ± 0.9	
	>60	43	3.26 ± 0.87	
Denial	18-30	173	2.83 ± 0.95	< .0001*
	31-40	112	3.66 ± 0.86	
	41-50	86	3.97 ± 0.78	
	51-60	84	3.42 ± 0.88	
	>60	43	3.4 ± 0.88	
Substance use	18-30	173	2.7 ± 0.94	< .0001*
	31-40	112	3.64 ± 0.97	
	41-50	86	3.81 ± 0.86	
	51-60	84	3.36 ± 0.93	
	>60	43	3.17 ± 1.06	
Use of emotional support	18-30	173	2.79 ± 0.94	< .0001*
	31-40	112	3.54 ± 0.91	
	41-50	86	3.78 ± 0.92	
	51-60	84	3.36 ± 0.93	
	>60	43	3.27 ± 0.95	
Use of instrumental support	18-30	173	2.71 ± 0.9	< .0001*
	31-40	112	3.65 ± 0.91	
	41-50	86	3.81 ± 0.91	
	51-60	84	3.45 ± 0.96	
	>60	43	3.34 ± 0.9	
Behavioral disengagement	18-30	173	2.76 ± 0.93	< .0001*
	31-40	112	3.5 ± 0.99	
	41-50	86	3.74 ± 0.84	
	51-60	84	3.38 ± 0.95	
	>60	43	3.26 ± 0.84	
Venting	18-30	173	2.67 ± 0.91	< .0001*
	31-40	112	3.57 ± 0.84	
	41-50	86	3.66 ± 0.79	
	51-60	84	3.33 ± 0.89	
	>60	43	3.35 ± 0.77	
Positive reframing	18-30	173	2.8 ± 0.96	< .0001*
	31-40	112	3.65 ± 0.85	
	41-50	86	3.82 ± 0.84	
	51-60	84	3.43 ± 0.93	
	>60	43	3.34 ± 0.89	
Planning	18-30	173	2.78 ± 0.9	< .0001*
	31-40	112	3.57 ± 0.95	
	41-50	86	3.84 ± 0.88	
	51-60	84	3.29 ± 0.86	
	>60	43	3.33 ± 0.92	
Humor	18-30	173	2.72 ± 0.94	< .0001*
	31-40	112	3.55 ± 0.96	
	41-50	86	3.78 ± 0.89	
	51-60	84	3.39 ± 1.02	
	>60	43	3.33 ± 1.01	
Acceptance	18-30	173	2.7 ± 0.93	< .0001*
	31-40	112	3.5 ± 0.91	
	41-50	86	3.7 ± 0.81	
	51-60	84	3.38 ± 0.93	
	>60	43	3.4 ± 0.95	
Religion	18-30	173	2.76 ± 1.03	< .0001*
	31-40	112	3.5 ± 0.94	
	41-50	86	3.81 ± 0.88	
	51-60	84	3.45 ± 0.86	
	>60	43	3.24 ± 0.92	
Self-blame	18-30	173	2.95 ± 0.94	< .0001*
	31-40	112	3.58 ± 0.94	
	41-50	86	3.82 ± 0.87	
	51-60	84	3.3 ± 0.98	
	>60	43	3.15 ± 0.88	
Physical health	18-30	173	2.82 ± 0.72	< .0001*
	31-40	112	3.57 ± 0.69	
	41-50	86	3.87 ± 0.63	
	51-60	84	3.36 ± 0.75	
	>60	43	3.26 ± 0.71	
Psychological well-being	18-30	173	2.83 ± 0.75	< .0001*
	31-40	112	3.59 ± 0.72	
	41-50	86	3.81 ± 0.66	
	51-60	84	3.45 ± 0.77	
	>60	43	3.29 ± 0.75	
Social relationships	18-30	173	2.84 ± 0.81	< .0001*
	31-40	112	3.5 ± 0.85	
	41-50	86	3.73 ± 0.84	
	51-60	84	3.37 ± 0.79	
	>60	43	3.29 ± 0.74	
Environment	18-30	173	2.79 ± 0.69	< .0001*
	31-40	112	3.59 ± 0.68	
	41-50	86	3.82 ± 0.63	
	51-60	84	3.36 ± 0.79	
	>60	43	3.31 ± 0.71	

The one-way ANOVA of the data (Table 4) highlighted notable trends regarding the impact of educational attainment on long-term blood donors' perceptions of digital health tools in enhancing their QoL and psychological adjustment. Statistically significant differences were observed across various educational groups, with participants possessing higher levels of education consistently reporting more favorable perceptions. For instance, those with a Bachelor’s degree or higher demonstrated higher mean scores in most factors, such as active coping, use of emotional support, self-blame, physical health, and psychological well-being, suggesting a stronger positive response to digital health tools compared to those with lower educational levels. In contrast, participants with lower levels of education, such as uneducated or with primary/secondary education, tended to report the lowest scores across the majority of measures.

**Table 4:** One-way ANOVA results assessing difference between participants perceptions on the impact of digital health tools on QoL and psychological adjustments of long-term blood donors - based on education * Statistically significant difference at .05 confidence interval; SD: Standard deviation

Factors	Education	N	Mean ± SD	p-value
Self-distraction	Uneducated	73	2.97 ± 0.91	< .0001*
	Primary/Secondary education	103	3.03 ± 0.95	
	Diploma	136	2.97 ± 0.88	
	Bachelor’s degree	101	3.79 ± 0.85	
	Master’s degree or higher	85	4.01 ± 0.75	
Active coping	Uneducated	73	2.97 ± 0.87	< .0001*
	Primary/Secondary education	103	2.92 ± 0.91	
	Diploma	136	2.99 ± 0.88	
	Bachelor’s degree	101	3.71 ± 0.94	
	Master’s degree or higher	85	3.98 ± 0.71	
Denial	Uneducated	73	2.95 ± 0.95	< .0001*
	Primary/Secondary education	103	3.04 ± 0.94	
	Diploma	136	3.08 ± 0.86	
	Bachelor’s degree	101	3.79 ± 0.87	
	Master’s degree or higher	85	4.04 ± 0.81	
Substance use	Uneducated	73	3.05 ± 0.85	< .0001*
	Primary/Secondary education	103	2.84 ± 0.97	
	Diploma	136	2.85 ± 0.95	
	Bachelor’s degree	101	3.76 ± 0.98	
	Master’s degree or higher	85	3.99 ± 0.82	
Use of emotional support	Uneducated	73	2.93 ± 0.86	< .0001*
	Primary/Secondary education	103	2.91 ± 0.95	
	Diploma	136	2.93 ± 0.92	
	Bachelor’s degree	101	3.72 ± 0.89	
	Master’s degree or higher	85	3.99 ± 0.81	
Use of instrumental support	Uneducated	73	3.01 ± 0.95	< .0001*
	Primary/Secondary education	103	2.91 ± 0.9	
	Diploma	136	2.95 ± 0.94	
	Bachelor’s degree	101	3.85 ± 0.93	
	Master’s degree or higher	85	3.86 ± 0.88	
Behavioral disengagement	Uneducated	73	3.03 ± 0.79	< .0001*
	Primary/Secondary education	103	2.83 ± 0.88	
	Diploma	136	2.91 ± 1.02	
	Bachelor’s degree	101	3.75 ± 0.92	
	Master’s degree or higher	85	3.86 ± 0.79	
Venting	Uneducated	73	2.99 ± 0.8	< .0001*
	Primary/Secondary education	103	2.93 ± 0.86	
	Diploma	136	2.84 ± 0.86	
	Bachelor’s degree	101	3.63 ± 0.92	
	Master’s degree or higher	85	3.84 ± 0.85	
Positive reframing	Uneducated	73	2.98 ± 0.9	< .0001*
	Primary/Secondary education	103	2.85 ± 0.88	
	Diploma	136	3.03 ± 0.86	
	Bachelor’s degree	101	3.87 ± 0.89	
	Master’s degree or higher	85	3.99 ± 0.85	
Planning	Uneducated	73	2.9 ± 0.83	< .0001*
	Primary/Secondary education	103	2.96 ± 0.88	
	Diploma	136	2.91 ± 0.93	
	Bachelor’s degree	101	3.77 ± 0.87	
	Master’s degree or higher	85	3.96 ± 0.82	
Humor	Uneducated	73	2.93 ± 0.98	< .0001*
	Primary/Secondary education	103	2.81 ± 0.85	
	Diploma	136	2.9 ± 0.96	
	Bachelor’s degree	101	3.79 ± 0.95	
	Master’s degree or higher	85	4.02 ± 0.81	
Acceptance	Uneducated	73	2.85 ± 0.97	< .0001*
	Primary/Secondary education	103	2.9 ± 0.88	
	Diploma	136	2.96 ± 0.86	
	Bachelor’s degree	101	3.65 ± 0.95	
	Master’s degree or higher	85	3.87 ± 0.86	
Religion	Uneducated	73	2.99 ± 0.88	< .0001*
	Primary/Secondary education	103	2.92 ± 0.98	
	Diploma	136	2.88 ± 0.93	
	Bachelor’s degree	101	3.75 ± 0.91	
	Master’s degree or higher	85	3.97 ± 0.87	
Self-blame	Uneducated	73	2.92 ± 0.89	< .0001*
	Primary/Secondary education	103	2.96 ± 0.89	
	Diploma	136	2.98 ± 0.84	
	Bachelor’s degree	101	3.79 ± 0.9	
	Master’s degree or higher	85	4.09 ± 0.81	
Physical health	Uneducated	73	3.02 ± 0.69	< .0001*
	Primary/Secondary education	103	2.9 ± 0.64	
	Diploma	136	3.03 ± 0.72	
	Bachelor’s degree	101	3.72 ± 0.81	
	Master’s degree or higher	85	3.96 ± 0.52	
Psychological well-being	Uneducated	73	3.02 ± 0.77	< .0001*
	Primary/Secondary education	103	2.95 ± 0.67	
	Diploma	136	3.01 ± 0.72	
	Bachelor’s degree	101	3.79 ± 0.77	
	Master’s degree or higher	85	3.92 ± 0.63	
Social relationships	Uneducated	73	3.03 ± 0.73	< .0001*
	Primary/Secondary education	103	2.86 ± 0.81	
	Diploma	136	2.93 ± 0.71	
	Bachelor’s degree	101	3.71 ± 0.85	
	Master’s degree or higher	85	3.99 ± 0.65	
Environment	Uneducated	73	3.07 ± 0.66	< .0001*
	Primary/Secondary education	103	2.89 ± 0.66	
	Diploma	136	2.98 ± 0.69	
	Bachelor’s degree	101	3.77 ± 0.75	
	Master’s degree or higher	85	3.89 ± 0.64	

The t-test results (Table 5) revealed significant gender-based differences in the perceptions of long-term blood donors regarding the impact of digital health tools on their QoL and psychological adjustment. Across all factors, female individuals reported consistently higher mean scores compared to males, indicating that women perceive digital health tools as more beneficial in enhancing their QoL and psychological well-being. For example, women scored notably higher in factors like active coping, denial, substance use, use of emotional support, and self-blame, reflecting a more positive engagement with these tools.

**Table 5:** One-way ANOVA results assessing difference between participants' perceptions on the impact of digital health tools on QoL and psychological adjustments of long-term blood donors - based on gender * Statistically significant difference at .05 confidence interval

Factors	Gender	N	Mean ± SD	p-value
Self-distraction	Male	245	2.86 ± 0.89	< .0001*
	Female	253	3.78 ± 0.83	
Active coping	Male	245	2.8 ± 0.9	< .0001*
	Female	253	3.75 ± 0.79	
Denial	Male	245	2.89 ± 0.88	< .0001*
	Female	253	3.81 ± 0.85	
Substance use	Male	245	2.69 ± 0.94	< .0001*
	Female	253	3.81 ± 0.81	
Use of emotional support	Male	245	2.8 ± 0.92	< .0001*
	Female	253	3.72 ± 0.86	
Use of instrumental support	Male	245	2.77 ± 0.91	< .0001*
	Female	253	3.79 ± 0.84	
Behavioral disengagement	Male	245	2.73 ± 0.9	< .0001*
	Female	253	3.74 ± 0.82	
Venting	Male	245	2.76 ± 0.87	< .0001*
	Female	253	3.65 ± 0.82	
Positive reframing	Male	245	2.8 ± 0.91	< .0001*
	Female	253	3.82 ± 0.79	
Planning	Male	245	2.78 ± 0.88	< .0001*
	Female	253	3.75 ± 0.84	
Humor	Male	245	2.76 ± 0.93	< .0001*
	Female	253	3.74 ± 0.91	
Acceptance	Male	245	2.76 ± 0.89	< .0001*
	Female	253	3.68 ± 0.85	
Religion	Male	245	2.75 ± 0.92	< .0001*
	Female	253	3.77 ± 0.86	
Self-blame	Male	245	2.83 ± 0.89	< .0001*
	Female	253	3.79 ± 0.83	
Physical health	Male	245	2.82 ± 0.69	< .0001*
	Female	253	3.76 ± 0.62	
Psychological well-being	Male	245	2.8 ± 0.69	< .0001*
	Female	253	3.8 ± 0.62	
Social relationships	Male	245	2.8 ± 0.81	< .0001*
	Female	253	3.73 ± 0.68	
Environment	Male	245	2.8 ± 0.66	< .0001*
	Female	253	3.76 ± 0.62	

## Discussion

The results of this study reveal a generally positive perception among long-term blood donors in Saudi Arabia regarding the impact of digital health tools on their QoL and psychological adjustment. These findings align with previous research, which indicates that digital health tools such as mobile apps, wearable devices, and telemedicine can enhance users' well-being and provide vital support for chronic health management [23]. Specifically, our findings show that participants value these tools for stress management, health monitoring, and emotional support, similar to what other studies have observed in chronic disease management contexts [24-27]. The objective of this study, which was to investigate the impact of digital health tools in enhancing the QoL and psychological adjustment of long-term blood donors in Saudi Arabia, was assessed from different contexts.

Age-based differences in perceptions emerged, with older participants perceiving digital health tools as more beneficial than younger donors. This result could be linked to a greater reliance on health support and a heightened awareness of health-related issues in older populations, as observed in the literature [23]. Furthermore, younger blood donors may have different expectations from digital health tools, potentially finding them less useful for their specific needs. This finding suggests the need for age-tailored digital health interventions to maximize engagement and relevance across age groups, an approach recommended by similar studies addressing chronic health conditions [56,57]. Tailoring these tools to better suit the needs and preferences of younger blood donors might help bridge the gap and increase the overall effectiveness of digital health interventions. Furthermore, the significant statistical differences underscored the importance of considering age as a key factor in designing and evaluating digital health solutions, as the response to such tools may not be uniform across different demographic groups. This could inform future strategies for improving the psychological well-being and coping mechanisms of long-term blood donors through targeted digital health initiatives.

Gender differences in the perception of digital health tools were also observed, with female participants consistently reporting higher perceived benefits in terms of emotional and psychological support. This aligns with prior research showing that women may be more likely to engage with health management tools for emotional support and stress management than men [58-60]. This highlights the importance of gender-sensitive design in digital health tools, potentially increasing their appeal and impact by addressing specific user needs more effectively. Although digital health tools are often perceived as universally beneficial, our findings suggest that factors such as age, gender, and educational background significantly influence user perceptions and experiences. This aligns with the study by Al-Hajri et al., which emphasized the need for culturally and demographically relevant adaptations of digital health tools for diverse populations [20].

In the present study, the gender gap was particularly pronounced in self-distraction, behavioral disengagement, venting, and humor, with females again reporting higher mean scores, suggesting that women may experience greater emotional benefits or engagement with coping mechanisms facilitated by digital health interventions. On the other hand, male individuals tended to score lower on these factors, which could indicate differences in coping styles or willingness to use digital tools for emotional support. The results suggest that gender may influence how digital health tools are perceived and utilized, with women perhaps benefiting more in terms of emotional support and psychological adjustment. This could imply that digital health tools need to be adapted or tailored to account for these gender differences, potentially offering more targeted interventions for males to increase engagement and psychological benefit.

Educational differences further underscore the need for tailored interventions. Participants with higher educational levels reported greater benefits from digital health tools, perhaps reflecting better digital literacy and health awareness, as has been suggested in previous studies on health tool usability [61]. Participants with lower education levels might face barriers related to technology use or may be less aware of potential benefits, emphasizing the importance of user-friendly design and accessible health information for all educational levels. The findings from the present study suggest that education may play a significant role in how individuals perceive and utilize digital health interventions, possibly due to differences in digital literacy, health awareness, or the ability to engage with more complex health-related information. For example, those with a Master’s degree or higher consistently showed the highest mean scores in self-blame, substance use, and venting, which may reflect a better understanding of psychological health and coping mechanisms.

The study also highlights the limitations of current digital health tools in addressing the specific psychological needs of long-term blood donors. While tools for general stress management were beneficial, aspects such as substance use and behavioral disengagement were perceived as less impacted. This finding reflects gaps identified in the reviews by López et al. [62] and Lattie et al. [63], which noted that many existing digital tools inadequately address complex psychological needs or fail to provide personalized interventions for specific behavioral challenges. Addressing this gap could involve incorporating evidence-based psychological strategies within digital platforms, such as cognitive-behavioral components or motivational modules tailored to donor-specific stressors.

Study limitations and future work

This study has several limitations. First, the cross-sectional design restricts causal inferences regarding the impact of digital health tools on long-term blood donors' QoL and psychological adjustment. Additionally, reliance on self-reported data may introduce response bias, as participants' perceptions might not fully reflect the objective impact of these tools. The focus on Saudi Arabia limits the generalizability of the findings, as cultural factors, such as attitudes toward health technology and gender dynamics in healthcare usage, may have influenced participants' experiences. Moreover, the non-random purposive sampling limits generalizability. Future research should consider using a random sample and include diverse populations to enhance external validity. Longitudinal studies are needed to establish causal relationships, and further exploration of specific digital features and culturally tailored interventions could provide more targeted insights for improving donor retention and well-being.

Implications

The findings of this study contribute to the theoretical understanding of digital health’s role in enhancing QoL and psychological adjustment, extending previous research primarily focused on chronic disease management to the unique context of long-term blood donors. The results underscore the need to consider demographic factors such as age, gender, and education level in digital health research, as these factors significantly influence user engagement and perception of benefits. Practically, these insights suggest that healthcare providers and developers should prioritize the customization of digital health tools to better meet the diverse needs of long-term donors. Tailoring these tools to address specific psychological stressors, coping mechanisms, and cultural considerations can potentially improve user experience, engagement, and donor retention, supporting a more resilient and sustainable blood donation system.

## Conclusions

This study underscores the positive role of digital health tools in enhancing the QoL and psychological adjustment of long-term blood donors in Saudi Arabia. Findings indicate that these tools are perceived as beneficial for managing physical and psychological challenges, especially among older and more educated donors, highlighting the influence of demographic factors on engagement. The findings of the study indicate that digital tools can play a significant role in facilitating coping mechanisms, promoting emotional well-being, and encouraging donor retention. However, it is important to exercise caution in making causal claims due to the cross-sectional design, which limits the ability to establish temporal relationships.

While the data provide evidence for the effectiveness of digital health tools, areas such as the role of substance use and behavioral disengagement in coping strategies require further exploration. These factors may influence how donors engage with digital tools and should be studied in greater depth to understand their impact fully. Similarly, the influence of demographic variables such as age, gender, and socioeconomic status on the use and effectiveness of digital health tools was not exhaustively analyzed, leaving room for further investigation. Future research should focus on these dimensions to strengthen the link between the study’s results and its conclusions. Longitudinal studies examining the interplay between coping mechanisms, digital engagement, and demographic factors could provide more robust insights, allowing for tailored interventions to optimize the benefits of digital health tools for diverse donor populations.

## References

[1] Peng C, Goswami P, Bai G (2020). A literature review of current technologies on health data integration for patient-centered health management. Health Informatics J.

[2] Garg S, Williams NL, Ip A, Dicker AP (2018). Clinical integration of digital solutions in health care: an overview of the current landscape of digital technologies in cancer care. JCO Clin Cancer Inform.

[3] Baltaxe E, Czypionka T, Kraus M (2019). Digital health transformation of integrated care in europe: overarching analysis of 17 integrated care programs. J Med Internet Res.

[4] Auerbach AD (2019). Evaluating digital health tools-prospective, experimental, and real world. JAMA Intern Med.

[5] Elkefi S, Trapani D, Ryan S (2023). The role of digital health in supporting cancer patients' mental health and psychological well-being for a better quality of life: a systematic literature review. Int J Med Inform.

[6] Victoria-Castro AM, Martin ML, Yamamoto Y (2024). Impact of digital health technology on quality of life in patients with heart failure. JACC Heart Fail.

[7] Su CC, Guo SE, Kuo YW (2024). Effects of internet-based digital health interventions on the physical activity and quality of life of colorectal cancer survivors: a systematic review and meta-analysis. Support Care Cancer.

[8] Van Den Hurk K, Peffer K, Habets K (2017). Blood donors' physical characteristics are associated with pre- and post-donation symptoms - Donor InSight. Blood Transfus.

[9] Parati G, Pellegrini D, Torlasco C (2019). How digital health can be applied for preventing and managing hypertension. Curr Hypertens Rep.

[10] Hoogerwerf MD, Veldhuizen IJ, de Kort WL, Frings-Dresen MH, Sluiter JK (2017). Factors associated with psychological and physiological stress reactions to blood donation: a systematic review of the literature. Giving Blood: Donor Stress and Hemostasis. Don't Let Your Blood Run Cold.

[11] Teglkamp J, Handgaard L, Hansen T (2020). The donors perceived positive and negative effects of blood donation. Transfusion.

[12] Makowicz D, Dziubaszewska R, Lisowicz K, Makowicz N (2022). Impact of regular blood donation on the human body; donors’ perspective. Donors’ opinion on side effects of regular blood donation on human body. J Trans Med.

[13] Bonk VA, France CR, Taylor BK (2001). Distraction reduces self-reported physiological reactions to blood donation in novice donors with a blunting coping style. Psychosom Med.

[14] Nissen-Meyer LS, Seghatchian J (2019). Donor health assessment - when is blood donation safe?. Transfus Apher Sci.

[15] France C, France France, JL JL (2020). Blood donation. Encyclopedia of Behavioral Medicine.

[16] Smıth A, Matthews R, Fıddler J (2011). Blood donation and community: exploring the influence of social capital. Int J Soc Inq.

[17] Schröder JM, Merz EM, Suanet B, Wiepking P (2023). The social contagion of prosocial behaviour: How neighbourhood blood donations influence individual donation behaviour. Health Place.

[18] Alsughayyir J, Almalki Y, Alalshaik M, Aljoni I, Kandel M, Alfhili MA, Alabdullateef A (2022). Demography and blood donation trends in Saudi Arabia: a nationwide retrospective, cross-sectional study. Saudi J Biol Sci.

[19] Abdelgader AM, Al Ghumlas AK (2020). The future of voluntary blood donation in the Kingdom of Saudi Arabia. Transfusion.

[20] Al-Hajri QR, Alfayez A, Alsalman D (2021). The impact of WhatsApp on the blood donation process in Saudi Arabia. J Blood Med.

[21] Syed W, Alsadoun A, Bashatah AS, Al-Rawi MB, Siddiqui N (2022). Assessment of the knowledge beliefs and associated factors among Saudi adults towards blood donation in Saudi Arabia. Hematology.

[22] Batis AA, Albarrak A (2021). Preferences and features of a blood donation smartphone app: a multicenter mixed-methods study in Riyadh, Saudi Arabia. Comp Method Prog Biomed Update.

[23] Debon R, Coleone JD, Bellei EA, De Marchi AC (2019). Mobile health applications for chronic diseases: a systematic review of features for lifestyle improvement. Diabetes Metab Syndr.

[24] Parrilla M, De Wael K (2021). Wearable Self‐Powered Electrochemical Devices for Continuous Health Management. Advance Funct Mat.

[25] Lu L, Zhang J, Xie Y, Gao F, Xu S, Wu X, Ye Z (2020). Wearable health devices in health care: narrative systematic review. JMIR Mhealth Uhealth.

[26] Lee SM, Lee D (2020). Healthcare wearable devices: an analysis of key factors for continuous use intention. Serv Bus.

[27] Poongodi T, Krishnamurthi R, Indrakumari R, Suresh P, Balusamy B (2020). Wearable devices and IoT. A Handbook of Internet of Things in Biomedical and Cyber Physical System.

[28] Niklas N, Loimayr C, Lenz J (2023). The impact of digital transformation on blood donation and donor characteristics. Transfus Med Hemother.

[29] Torrent-Sellens J, Salazar-Concha C, Ficapal-Cusí P, Saigí-Rubió F (2021). Using digital platforms to promote blood donation: motivational and preliminary evidence from Latin America and Spain. Int J Environ Res Public Health.

[30] Hoogerwerf MD, Veldhuizen IJ, De Kort WL, Frings-Dresen MH, Sluiter JK (2015). Factors associated with psychological and physiological stress reactions to blood donation: a systematic review of the literature. Blood Transfus.

[31] Ray S, Singh Z, Banerjee A (2005). Psychosocial variables of voluntary blood donors at blood bank of a medical college. Med J Armed Forces India.

[32] Merz EM, Ferguson E, van Dongen A (2018). Psychosocial characteristics of blood donors influence their voluntary nonmedical lapse. Transfusion.

[33] Kaloupek DG, White H, Wong M (1984). Multiple assessment of coping strategies used by volunteer blood donors: implications for preparatory training. J Behav Med.

[34] Eysenck MW (2007). Cognition and stress. Encyclopedia of Stress (Second Edition).

[35] Monteiro TH, Ferreira ÍJD, Junior AC, Chocair HS, Ferreira JD (2024). Barriers and motivations for blood donation: an integrative review. Hematol Transfus Cell Ther.

[36] van Olmen J (2022). The promise of digital self-management: a reflection about the effects of patient-targeted e-health tools on self-management and wellbeing. Int J Environ Res Public Health.

[37] Rickard N, Arjmand HA, Bakker D, Seabrook E (2016). Development of a mobile phone app to support self-monitoring of emotional well-being: a mental health digital innovation. JMIR Ment Health.

[38] Pal K, Dack C, Ross J (2018). Digital health interventions for adults with type 2 diabetes: qualitative study of patient perspectives on diabetes self-management education and support. J Med Internet Res.

[39] Espinoza Chamorro R, Santos LH, Mori Y, Liu C, Yamamoto G, Kuroda T (2024). Gamification approach to provide support about the deferral experience in blood donation: design and feasibility study. JMIR Hum Factors.

[40] Savioli ML, Sakashita AM, Cipolletta AN, Brandão RC, Kutner JM (2024). Telemedicine pre-screening for blood donor [In Press]. Hematol Transfus Cell Ther.

[41] (2023). world Health Organization: Blood safety and availability. Blood safety and availability [Internet.

[42] (2024). American Red Cross: Requirements by donation types. https://www.redcrossblood.org/donate-blood/how-to-donate/eligibility-requirements.html#:~:text=Power%20Red%20Donation%20*%20Donation%20frequency:%20Every,tall%20and%20weigh%20at%20least%20150%20lbs.

[43] Thorpe R, Masser B, Coundouris SP, Hyde MK, Kruse SP, Davison TE (2024). The health impacts of blood donation: a systematic review of donor and non-donor perceptions. Blood Transfus.

[44] Bosnes V, Aldrin M, Heier HE (2005). Predicting blood donor arrival. Transfusion.

[45] Bani-Ahmad MA, Khabour OF, Gharibeh MY, Alshlool KN (2017). The impact of multiple blood donations on the risk of cardiovascular diseases: Insight of lipid profile. Transfus Clin Biol.

[46] Krebs P, Duncan DT (2015). Health app use among US mobile phone owners: a national survey. JMIR Mhealth Uhealth.

[47] Peng W, Kanthawala S, Yuan S, Hussain SA (2016). A qualitative study of user perceptions of mobile health apps. BMC Public Health.

[48] Suryani A, Herianti Eva (2023). Purposive sampling and ordinary least square analysis: investigating the relationship between managerial overconfidence, transfer pricing and tax management in Indonesian stock exchange-listed firms. Int J Prof Bus Rev.

[49] Palinkas LA, Horwitz SM, Green CA, Wisdom JP, Duan N, Hoagwood K (2015). Purposeful sampling for qualitative data collection and analysis in mixed method implementation research. Adm Policy Ment Health.

[50] Etikan I, Musa SA, Alkassim RS (2015). Comparison of convenience sampling and purposive sampling. Am J Theoretical App Stat.

[51] Sarmah HK, Hazarika BB, Choudhury G (2013). An investigation on effect of bias on determination of sample size on the basis of data related to the students of schools of Guwahati. Int J App Math Stat Sci.

[52] Casamali FF, Schuch FB, Scortegagna SA, Legnani E, De Marchi AC (2019). Accordance and reproducibility of the electronic version of the WHOQOL-BREF and WHOQOL-OLD questionnaires. Exp Gerontol.

[53] Rawlings GH, Thompson AR, Armstrong I, Novakova B, Beail N (2022). Coping styles associated with depression, health anxiety and health-related quality of life in pulmonary hypertension: cross-sectional analysis. BMJ Open.

[54] Beaton DE, Bombardier C, Guillemin F, Ferraz MB (2000). Guidelines for the process of cross-cultural adaptation of self-report measures. Spine (Phila Pa 1976).

[55] Taber KS (2018). The use of Cronbach’s alpha when developing and reporting research instruments in science education. Res Sci Educ.

[56] Whitehead L, Robinson S, Arabiat D, Jenkins M, Morelius E (2024). The report of access and engagement with digital health interventions among children and young people: systematic review. JMIR Pediatr Parent.

[57] (2020). United Nations Educational, Scientific and Cultural Organization. Youth-centred digital health interventions [Internet]. World Health Organization. Youth-centred Digital Health Interventions: A Framework for Planning, Developing and Implementing Solutions With and for Young People.

[58] Graves BS, Hall ME, Dias-Karch C, Haischer MH, Apter C (2021). Gender differences in perceived stress and coping among college students. PLoS One.

[59] Bidmon S, Terlutter R (2015). Gender differences in searching for health information on the internet and the virtual patient-physician relationship in Germany: exploratory results on how men and women differ and why. J Med Internet Res.

[60] Balaskas A, Schueller SM, Doherty K, Cox AL, Doherty G (2024). Designing personalized mental health interventions for anxiety: CBT therapists’ perspective. Int J Hum Comput Stud.

[61] (2018). Frost and Sullivan: Femtech—time for a digital revolution in the women’s health market. https://www.frost.com/growth-opportunity-news/femtechtime-digital-revolution-womens-health-market/.

[62] Arias López MD, Ong BA, Borrat Frigola X (2023). Digital literacy as a new determinant of health: a scoping review. PLOS Digit Health.

[63] Lattie EG, Stiles-Shields C, Graham AK (2022). An overview of and recommendations for more accessible digital mental health services. Nat Rev Psychol.

